# B Chromosome Transcriptional Inactivation in the Spermatogenesis of the Grasshopper *Eyprepocnemis plorans*

**DOI:** 10.3390/genes15121512

**Published:** 2024-11-25

**Authors:** Juan Luis Santos, María Teresa Parra, Sara Arévalo, Andrea Guajardo-Grence, Jesús Page, José Ángel Suja, Carlos García de la Vega, Alberto Viera

**Affiliations:** 1 Departamento de Genética, Facultad de Biología, Universidad Complutense de Madrid, 28040 Madrid, Spain; jlsc53@bio.ucm.es; 2 Departamento de Biología, Universidad Autónoma de Madrid, 28049 Madrid, Spain; mayte.parra@uam.es (M.T.P.); sara.arevalom@estudiante.uam.es (S.A.); andrea.guajardo@uam.es (A.G.-G.); jesus.page@uam.es (J.P.); jose.suja@uam.es (J.Á.S.); carlos.delavega@uam.es (C.G.d.l.V.)

**Keywords:** sex chromosomes, B chromosomes, meiosis, transcriptional activity, grasshopper, histone modifications, *Eyprepocnemis plorans*

## Abstract

Background/Objectives: We analyzed the relationship between synapsis, recombination, and transcription during the spermatogenesis of the grasshopper *Eyprepocnemis plorans* carrying B chromosomes (type B1). Methods: The progression of synapsis was interpreted according to the dynamics of the cohesin subunit SMC3 axes. DNA double-strand breaks were revealed by RAD51 immunolabeling, while transcriptional activity was determined by the presence of RNA polymerase II phosphorylated at serine 2 (pRNApol II) immunolabeling. The two repressive epigenetic modifications, histone H3 methylated at lysine 9 (H3K9me3) and histone H2AX phosphorylated at serine 139 (γ-H2AX), were employed to reveal transcriptional inactivity. Results: During prophase I, spermatocytes with one B1 chromosome showed overall transcription except in the regions occupied by both the X and the B1 chromosomes. This transcriptional inactivity was accompanied by the accumulation of repressive epigenetic modifications. When two B1 chromosomes were present, they could appear as a fully synapsed monochiasmatic bivalent, showing intense H3K9me3 labeling and absence of pRNApol II, while γ-H2AX labeling was similar to that shown by the autosomes. Conclusions: According to our results, B1 transcriptional inactivation was triggered in spermatogonia, long before the beginning of meiosis, and was accompanied by H3K9me3 heterochromatinization that was maintained throughout spermatogenesis. Moreover, when two B1 were present, the transcriptional inactivation did not preclude synapsis and recombination achievement by these chromosomes.

## 1. Introduction

Progression of synapsis and recombination of homologous chromosomes during prophase I of meiosis are accompanied by changes in chromatin organization [1,2], the presence of certain epigenetic marks [3,4,5,6] and the reactivation of transcriptional activity [7,8,9,10,11]. In mammals, the rate of transcription in spermatogonia decreases as meiosis initiates and remains at low levels during synapsis progression (leptotene and zygotene stages) and is reactivated when full synapsis is achieved (pachytene stage) [8,9,10,12,13]. However, chromosomal regions that fail to complete synapsis are inactivated undergoing a process denominated meiotic silencing of unsynapsed chromatin (MSUC) [14,15,16]. Thus, there is no reactivation of transcription in unsynapsed chromosomes and meiotic arrest can take place. In the unsynapsed regions of the mammalian sex chromosomes (X and Y), this process is especially conspicuous and is denominated meiotic sex chromosome inactivation (MSCI) [17,18,19]. Therefore, MSUC and MSCI are intimately related to the progression of DNA recombination and the achievement of pairing and synapsis of homologous chromosomes during prophase I. On the other hand, species of other taxa display deviations from the above-described pattern of the regulation of transcription and MSUC/MSCI. For instance, the autosomes of grasshoppers, moths, true bugs and scorpions present intense transcriptional activity during early prophase I stages [20,21,22,23,24,25,26,27,28,29].

In previous work, we showed that in *E. plorans* males, which only present a single X sex chromosome that behaves as an asynaptic and achiasmatic univalent during meiosis, transcriptional activity is maintained in most autosomal regions from leptotene to diakinesis, while the single X chromosome is invariably inactive [28]. This species is characterized by an extensive polymorphism for distinct types of B chromosomes [30,31,32]. B chromosomes are dispensable extra chromosomes that do not recombine with the standard chromosomes and thus follow their own evolutionary pathway [33]. Here, we analyzed the transcriptional activity of the B1 chromosome during spermatogenesis. For this purpose, we checked the distribution of pRNApol II and two heterochromatin markers (H3K9me3 and γ-H2AX), as well as a protein involved in meiotic recombination (RAD51), in males with different B numbers.

## 2. Materials and Methods

Adult males of *E. plorans* (Orthoptera: Acrididae) collected in a natural population from San Juan de Alicante (Spain), under the appropriated licenses and conditions (authorization #3126/nm from Conselleria de Agricultura, Medio Ambiente, Cambio Climático y Desarrolllo Rural; Generalitat Valenciana), were used for this study. *E. plorans* karyotype is composed of 22 autosomes, the chromosomal sex system being of the type XX in the females and X0 in the males. The presence of supernumerary B1 chromosomes was checked in testicular biopsies fixed in 3:1 ethanol/glacial acetic acid and squashed in a drop of 50% glacial acetic acid. For each male, at least 20 diplotene/metaphase I cells were analyzed under phase contrast microscopy to determine the number of B1 chromosomes. Accordingly, we classified the individuals as 0B (without B1 chromosomes, n = 10), 1B (with one B1 chromosome, n = 15), and 2B (with two B1 chromosomes, n = 15). The number of heterochromatic bodies, representing the X and B1 chromosomes, found in leptotene, zygotene and pachytene nuclei was analyzed in 120 cells per stage from three different individuals of each chromosome constitution, 0, 1 and 2 B1 chromosomes, after C-banding and DAPI staining. Additionally, we determined the number of metaphase I cells (n = 120) presenting a B1 bivalent in individuals presenting two B1 chromosomes.

### 2.1. Spreading and Squashing of Seminiferous Tubules

The animals were euthanized and dissected in PBS (137 mM NaCl, 2.7 mM KCl, 10.1 mM Na_2_HPO_4_, 1.7 mM KH_2_PO_4_, pH 7.4) and the testes removed. Subsequently, the testes were cleaned in PBS and seminiferous tubules were processed for either spreading or squashing. The spreading of spermatocytes was performed following a drying-down technique [34] with slight modifications. Briefly, the proximal region of tubules was removed with an entomological pin to eliminate most mature spermatids and spermatozoa. These tubules were homogenized in 100 mM sucrose in distilled water and macerated for 15 min at room temperature. Spermatocytes were simultaneously spread onto a slide and fixed with 1% paraformaldehyde in distilled water containing 0.15% Triton X-100. The slides were then left to dry for 2 h in a moist chamber, and finally washed with 0.08% Photo-Flo (Kodak, Rochester, NY, USA) in distilled water and air-dried. Squashing of tubules was performed as previously described [35]. Briefly, seminiferous tubules were fixed in freshly prepared 2% formaldehyde in PBS containing 0.1% Triton X-100 (Sigma, Marlborough, MA, USA). After 5 min, several fragments were placed on a slide previously coated with 1 mg/mL poly-L-lysine (Sigma, Marlborough, MA, USA) with a drop of fixative and minced with tweezers. After squashing and freezing in liquid nitrogen, the coverslip was removed.

We combined squashing and spreading techniques as the most adequate methodologies to achieve the purposes of this work. In squashed spermatocytes, the nuclear volume and chromatin organization were not disturbed, allowing for a precise study of 3D distribution of different structures within a cell. Moreover, X and B1 chromosomes could be identified as positive heteropycnotic in both squashed prophase I spermatocytes and early spermatids stained with DAPI. In contrast, spread spermatocytes were projected into a single focal plane, facilitating their visualization, but the X and B1 chromosomes could not be identified after DAPI staining due to chromatin spreading.

### 2.2. Immunofluorescence Microscopy

Immunofluorescence experiments were carried out in parallel in individuals bearing 0, 1 and 2 B1 chromosomes in at least two preparations per individual. These experiments were replicated at least three times in different individuals to exclude differences among either individuals or antibodies, and more than 100 cells were analyzed per stage. Preparations of seminiferous tubules were incubated only with secondary antibodies to exclude the possibility of cross-reaction with spermatocytes. It must be noted that the antibodies used in this study were previously employed in several orthopteran species, including *E. plorans* [28,36], heteropteran species [29], scorpions [26], or the *Ephestia kuehniella* [27] and *Bombyx mori* [37]. Spread or squashed preparations were rinsed three times for 5 min in PBS and incubated overnight at 4 °C with the corresponding primary antibodies diluted in PBS. Cohesin subunit SMC3 was detected using the K987 rabbit polyclonal antisera kindly provided by Dr. Barbero [36,38], raised against a synthetic peptide corresponding to the carboxy-terminal amino-acid sequence of human SMC3, at 1:30 dilution. A rabbit polyclonal anti-RAD51 antibody (Oncogene Research Products, La Jolla, CA, USA, Ab-1, PC130), generated against recombinant HsRad51 protein, was used at 1:50 dilution. To detect γ-H2AX, we used a monoclonal mouse antibody (Upstate, Burlington, MA, USA, 05-636) at 1:1000 dilution. Histone H3 trimethylated at lysine 9 (H3K9me3) was revealed with a rabbit polyclonal serum (Abcam, Cambridge, UK, ab-8898) at 1:100 dilution. RNA polymerase II was revealed with a mouse monoclonal antibody anti-RNA polymerase II phosphorylated at serine 2 (Abcam, Cambridge, UK, ab-24758) at 1:100 dilution. Following three washes in PBS, the slides were incubated for 30 min at room temperature with the corresponding secondary antibodies at 1:100 dilution. The secondary antibodies used were donkey anti-rabbit IgG (Molecular Probes, ThermoFisher Scientific, Rockford, IL, USA) and donkey anti-mouse IgG (Molecular Probes, ThermoFisher Scientific, Rockford, IL, USA), conjugated with Alexa 488 or Alexa 594, and a donkey anti-mouse IgM conjugated with DyLight 594 (Jackson ImmunoResearch Laboratories, West Grove, PE, USA). The slides were subsequently rinsed in PBS and counterstained for 3 min with 5 μg/mL DAPI. After a final rinse in PBS, the slides were mounted in Vectashield (Vector Laboratories, Burlingame, CA, USA) and sealed with nail polish. Double-immunolabeling experiments, in which the two primary antibodies were generated in the same host species (SMC3 with either RAD51 or H3K9me3), were performed as previously described [39]. In these cases, the slides were firstly incubated with the anti-SMC3 antibody for 1 h at room temperature, rinsed three times for 5 min in PBS and incubated overnight at 4 °C with a DyLight 488-conjugated goat Fab′ fragment anti-rabbit IgG (Jackson ImmunoResearch Laboratories, West Grove, PE, USA) at 1:100 dilution in PBS. Afterwards, the slides were rinsed six times for 5 min in PBS, incubated with anti-RAD51 or anti-H3K9me3 for 1 h, rinsed three times for 5 min in PBS, and then incubated with Alexa 594 donkey anti-rabbit IgG (Molecular Probes, ThermoFisher Scientific, Rockford, IL, USA) at 1:100 dilution.

### 2.3. Image Acquisition and Processing

Observations were performed using an Olympus BX61 microscope (Olympus, Hamburg, Germany) equipped with a motorized Z axis and epifluorescence optics. Single images or image stacks were captured with an Olympus DP71 digital camera controlled by CellF Imaging System (Münster, Germany) under fixed capture conditions to facilitate the comparison among signal intensity. The images were analyzed and processed using the public domain ImageJ software (National Institutes of Health, Bethesda, MD, USA; https://imagej.net accessed on 16 November 2024. Final figures were processed with Adobe Photoshop 7.0 or CS5 software (Adobe Systems Inc., San Jose, CA, USA).

### 2.4. RAD51 Foci Quantification

We determined the number of RAD51 foci in ten zygotene and pachytene nuclei from three different individuals presenting two B1 chromosomes. RAD51 foci were scored using the program ImageJ 1.53t, “Analyse particles” function. For statistical analyses, unpaired two-tailed *t*-test were performed to compare RAD51 foci associated with the B1 or autosomal bivalents. The data are presented by a box whiskers plot using GraphPad Prism 6.0 software (Boston, MA, USA).

### 2.5. Fluorescence Quantification

For the quantification of DAPI, pRNApol II or H3K9me3 fluorescence intensity in a single nucleus, we calculated the integrated density of fluorescence using ImageJ in images captured under fixed conditions. To compare the relative fluorescence intensity of DAPI and pRNApol II labelings at the X and B1 chromosomes or autosomes, we proceeded as follows. We analyzed zygotene (n = 5) and pachytene (n = 5) spread nuclei from individuals presenting 0, 1 and 2 B1 chromosomes. We empirically selected the nuclear region occupied by the X chromosome (in 0B1 individuals), the X and B1 chromosomes (in 1B1 individuals) or by the X and two B1 chromosomes (2B1 individuals) in a given nucleus. DAPI and pRNApol I staining integrated densities were measured and afterwards the same selection was placed over the nuclear region occupied by autosomes to repeat the measurements. Three background fluorescence measurements were acquired in the same image. The corrected total fluorescence was calculated as follows: corrected total fluorescence = integrated density of fluorescence—(area of selection X mean fluorescence of background readings). Finally, to facilitate comparisons and their representation, we determined the intensity ratio between pRNApol II/DAPI labeling found over X/B1 chromosomes and autosomes in each analyzed cell. For the quantification of H3K9me3 fluorescence intensity in squashed spermatogonial cells from individuals bearing 0 (n = 18), 1 (n = 18) and 2 B1 chromosomes (n = 18), we initially obtained the projections of complete nuclei. A binary mask was generated from the DAPI staining, achieving a measurement for the nuclear area and we proceeded as mentioned above. For statistical analyses, an ANOVA test (confidence level: 95%) and a Tukey’s multiple comparisons test were performed. Data were presented by a scatter dot plot using GraphPad Prism 6.0 software (Boston, MA, USA).

### 2.6. Electron Microscopy

In order to observe synaptonemal complexes, surface spreading was performed according to the work of del Cerro and Santos [40]. Briefly, seminiferous tubules were disaggregated in Dulbecco’s medium (Gibco, 4500 mg D-glucose/L with L glutamine and without Na pyruvate) containing 2 mM EDTA and 0.1% bovine serum albumin. On a cavity slide, spreading medium containing 60 mM NaPO_4_ buffer, 1 mM EDTA, 0.03% Triton X-100 (pH 7.5) was added to a drop of seminiferous tubules suspension. After incubating for 10 min, the suspension was added onto plastic-coated slides with 350 µL of paraformaldehyde fixative (pH 8.9). Preparations were dried overnight in a moist chamber at 35 °C, rinsed in distilled water and air-dried. For silver impregnation, 100 µL of 33% AgNO_3_ was added to each preparation, which was covered with a piece of nylon cloth. Fifteen pachytene spermatocytes were examined and photographed using a transmission Jeol JM1010 electron microscope (Tokyo, Japan).

## 3. Results

### 3.1. Meiotic Behavior of the B1 Chromosome

The karyotype of *E. plorans* is composed of 23 and 24 acrocentric chromosomes: 22 + X in the males and 22 + XX in the females. In addition, individuals from distinct populations frequently show a variable number of different supernumerary B chromosomes that have been classified according to their size, morphology, and C-banding patterns. The B1 chromosome was the first variant described and is predominant in most of the natural Spanish populations analyzed [30,31].

The meiotic behavior of the single B1 chromosome is similar to that of the X chromosome. Briefly, (i) both exhibit positive heteropycnosis and asynapsis during prophase I, (ii) both preserve the univalent condition until metaphase I, and (iii) both show reductional segregation at anaphase I and equational segregation at anaphase II. However, when two B1 chromosomes are present in the same spermatocyte, they can form a stable bivalent and segregate as autosomes at anaphases I and II (Supplementary Figure S1).

### 3.2. Cohesin Subunit SMC3 Immunolabeling as a Marker for Chromosome Pairing and Synapsis Throughout Prophase I Stages

**Figure 1:**
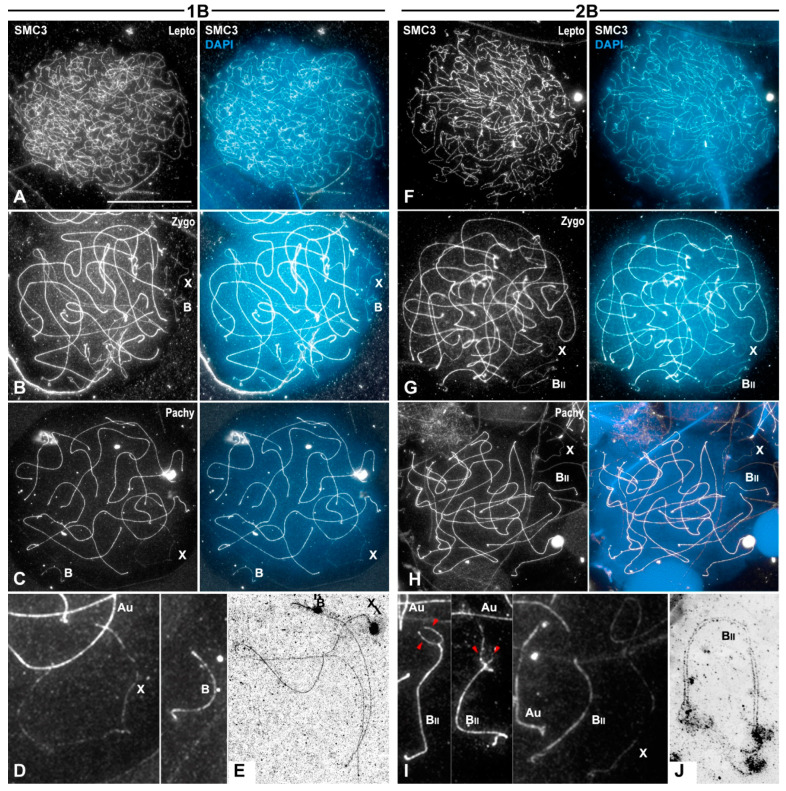
Dynamics of the B1 univalent and bivalent revealed by SMC3 immunolabeling. Prophase I spread spermatocytes from individuals with a B1 univalent (**A**–**E**) or a B1 bivalent (**F**–**J**). The positions of the sex chromosome (X), the B1 univalent (B), the B1 bivalent (B_II_) and of autosomal bivalents (Au) are indicated. Nuclei are counterstained with DAPI (blue). (**A**) At leptotene, SMC3 axes (white) are present all along the chromosomes. Thus, the identification of the X and B1 chromosomes was impeded by the imbrication of the SMC3 axes. (**B**) During zygotene, SMC3 autosomal axes were gradually paired into thicker filaments except at the X and B1 chromosomes. (**C**) At pachytene, autosomal bivalents displayed fully paired SMC3 axes, whereas a single thin SMC3 axis was observed in the X and B1 chromosomes. (**D**) 300% magnification of the nuclear region occupied by the X and B1 chromosomes. (**E**) Electron microscopy microphotograph of a pachytene spermatocyte showing the unsynapsed X and B1 chromosomes. (**F**) Leptotene spermatocytes with thin SMC3 axes all over the nucleus. (**G**) During zygotene, the SMC3 axes of the B1 chromosomes were gradually paired, as in the case of autosomes. (**H**) Pachytene spermatocyte showing fully paired SMC3 axes all along the autosomal bivalents, whereas pairing delay was observable in one of the ends of the B1 bivalent. (**I**) 300% magnifications of selected B1 bivalents with unpaired ends (red arrowheads), or with complete paired SMC3 axes as the autosomes (Au). In contrast, the X univalent exhibited a single unpaired SMC3 axis. (**J**) Electron microscopy microphotograph of a pachytene spermatocyte with fully synapsed B1 bivalent. Bar in (**A**), 10 µm.

Previous studies have shown that the dynamics of the cohesin subunit SMC3 axes onto grasshopper spermatocytes allow for the determination of meiotic stages [38,41]. At leptotene, SMC3 axes of all chromosomes were imbricated and unpaired. Thus, the single B1 chromosome could not be undoubtedly identified within the nucleus (Figure 1A). At zygotene, autosomes progressively undergo pairing. In those paired segments, SMC3 axes appear as thicker filaments (Figure 1B). The single X and B1 chromosomes could be identified since both showed thin unpaired SMC3 axes (Figure 1B). At pachytene, all autosomal bivalents presented thick SMC3 axes along their full length, while two thin SMC3 axes clearly identified the asynaptic condition of both the X and the B1 chromosomes (Figure 1C,D). Electron microscope observations confirmed these results (Figure 1E).

In the case of individuals with two B1 chromosomes, SMC3 labeling of these chromosomes during prophase I was similar to that shown by the autosomes (Figure 1F–H), although the synapsis of B1 chromosomes was delayed (Figure 1H,I). Synapsis fulfilment by the B1 chromosomes could be inferred by the complete pairing of their SMC3 axes (Figure 1I) and by observations in electron microscopy (Figure 1J).

### 3.3. B1 Bivalent Is Chiasmatic

**Figure 2:**
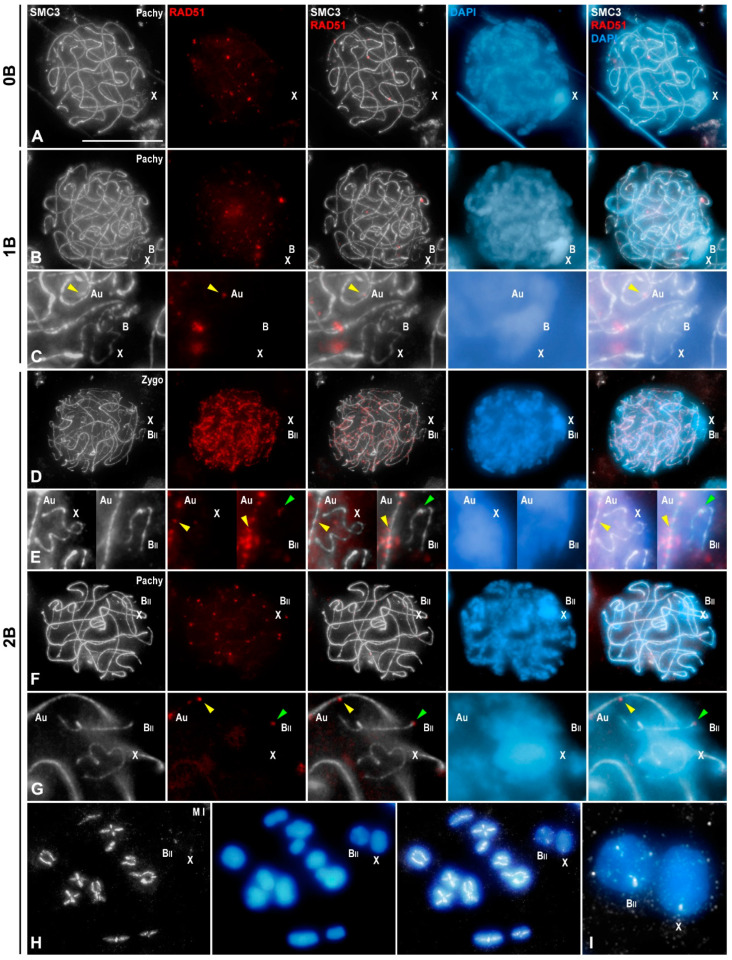
Meiotic reciprocal recombination in B1 chromosomes. Double immunolabeling of SMC3 (white) and RAD51 (red) in squashed spermatocytes counterstained with DAPI (blue) in individuals without B1 chromosomes (0B) and bearing one B1 (1B) or two B1 chromosomes (2B). The positions of the sex chromosome (X), the B1 univalent (B), the B1 bivalent (B_II_) and autosomal bivalents (Au) are indicated. Note that in prophase I stages, the X and B1 chromosomes are heteropycnotic positive chromatin bodies after DAPI staining. (**A**) Pachytene spermatocyte of a 0B male. RAD51 foci are visible along paired SMC3 axes of autosomes, but not over the X chromosome. (**B**) Pachytene spermatocyte of a 1B male. RAD51 foci are located over the fully paired SMC3 axes of autosomes but not over the unpaired SMC3 axes of the X and B1 chromosomes. (**C**) 300% magnification of the nuclear region occupied by the X and B1 chromosomes (yellow arrowheads signal RAD51 focus on an autosomal bivalent). (**D**) Zygotene spermatocyte of a 2B male. RAD51 foci were found over paired and unpaired SMC3 axes of autosomal bivalents and the B1 bivalent. (**E**) 300% magnification of the nuclear region occupied by the X chromosome and the B1 bivalent. Yellow and green arrowheads signal RAD51 foci located on autosomal and B1 bivalents, respectively. (**F**) Early/mid pachytene spermatocyte of a 2B male. Autosomal and B1 bivalents displayed RAD51 foci over their paired SMC3 axes. (**G**) 300% magnification of the nuclear region occupied by the X chromosome and the B1 bivalent. Yellow and green arrowheads signal RAD51 foci located on autosome and B bivalents, respectively. (**H**) Metaphase I spermatocyte of a 2B1 male. SMC3 appeared at the interchromatid domain of autosomal and B1 bivalents and was interrupted at chiasma sites. (**I**) 300% magnification of the X chromosome and the B1 bivalent. Bar in (**A**), 10 µm.

To determine whether two B1 chromosomes were able to arrange as a stable bivalent in which they formed a single chiasma, we initially estimated the number of heterochromatic bodies found after C-banding. In individuals without B1 chromosomes, we invariably observed a single heterochromatic body, corresponding to the X chromosome, in all the analyzed leptotene, zygotene and pachytene spermatocytes (120 cells were scored for each meiotic stage). Individuals bearing one B1 chromosome presented two heterochromatic bodies, the X and the B1 chromosomes, in 95.83%, 99.17% and 98.33% of leptotene, zygotene and pachytene spermatocytes, respectively (120 cells were scored for each meiotic stage). As regards individuals with two B1 chromosomes, we found the expected three heterochromatic bodies, namely, the X and two B1 chromosomes, in 97.5% of leptotene nuclei (n = 120). In contrast, 67.5% of zygotene nuclei (n = 120) presented only two heterochromatic bodies, the X and the B1 bivalent. This percentage raised in pachytene nuclei (n = 120) up to 90.83%. Likewise, we also found that 87.5% of metaphase I cells (n = 120) from individuals with two B1 chromosomes presented both chromosomes arranged into a bivalent. Moreover, we performed double immunolabeling of SMC3 and RAD51 onto squashed spermatocytes. The recombinase RAD51 is considered a useful marker for identifying the locations where double-strand breaks (DSBs) are repaired during meiosis [42]. As previously reported, *E. plorans* spermatocytes without B chromosomes presented RAD51 foci over the SMC3 axes of autosomes from leptotene up to pachytene. However, they were absent on the thin SMC3 axis of the unsynapsed X chromosome (Figure 2A) [38]. Similarly, in individuals with a single B1 chromosome, RAD51 labeling was present only on the autosomes (Figure 2B,C) [36]. However, in individuals with two B1 chromosomes, RAD51 labeling was detected in both B1 and autosomal bivalents in zygotene (Figure 2D,E) and pachytene spermatocytes (Figure 2F,G). The average number of RAD51 foci per autosomal bivalent was 30.17 in zygotene (n = 30) and 5.17 in pachytene (n = 30). In contrast, the B1 bivalent displayed 6.4 RAD51 foci in zygotene and 1.67 in pachytene, whereas this antibody was never detected over the X chromosome. Thus, the B1 bivalent always displayed a lower number of RAD51 foci than autosomal ones in zygotene (unpaired two-tailed *t*-test, *p* < 0.0001), and pachytene (unpaired two-tailed *t*-test, *p* < 0.0001) spermatocytes (Supplementary Graph S1A). At metaphase I, SMC3 axes of autosomal and B1 bivalents were continuous except at the regions where chiasmata occurred (Figure 2H,I). Taking into account these findings, we can conclude that the B1 bivalent is chiasmatic.

### 3.4. Transcriptional Activity of B1 Chromosomes Does Not Depend on Their Univalent or Bivalent Condition

**Figure 3:**
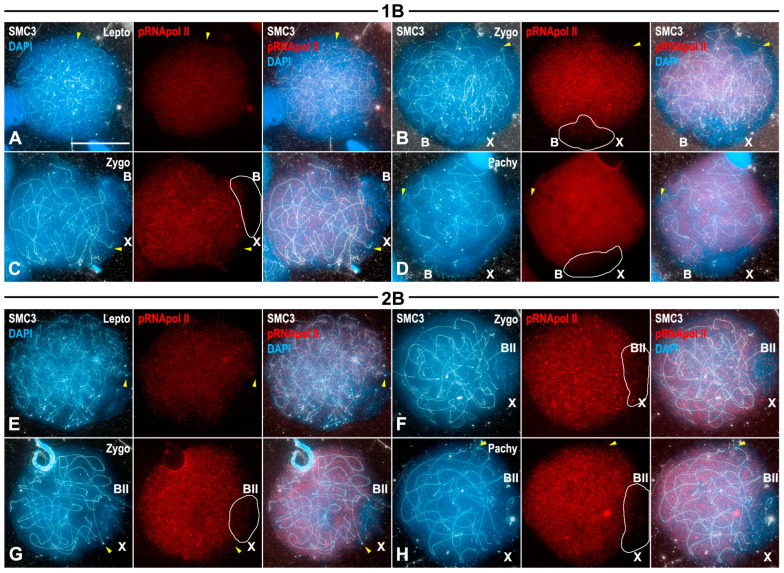
Transcriptional activity of B1 chromosomes during prophase I stages. Double immunolabelings of SMC3 (white) and pRNApol II (red) in prophase I spread spermatocytes counterstained with DAPI (blue) from individuals with one B1 (**A**–**D**) or two B1 chromosomes (**E**–**H**). The positions of the sex chromosome (X), the B1 univalent (B) and the B1 bivalent (B_II_) are indicated, and in (**B**–**D**,**F**–**H**), their positions are outlined. (**A**) In leptotene, pRNApol II labeling appeared spread throughout most of the nucleus although reduced at some regions (yellow arrowheads). (**B**–**D**) During zygotene (**B**,**C**) and pachytene (**D**), pRNApol II labeling covered most of the autosomal chromatin irrespectively of their pairing status. The labeling was clearly reduced only in particular regions possibly corresponding to chromosome ends (yellow arrowheads). pRNApol II labeling is absent in the regions occupied by both the X and the B1 univalents. (**E**,**F**) The pattern of the pRNApol II labeling in individuals with two B1 chromosomes is identical to that described above. Significantly, the region occupied by the B1s does not present any signal from leptotene up to pachytene. Bar in (**A**), 10 µm.

**Figure 4:**
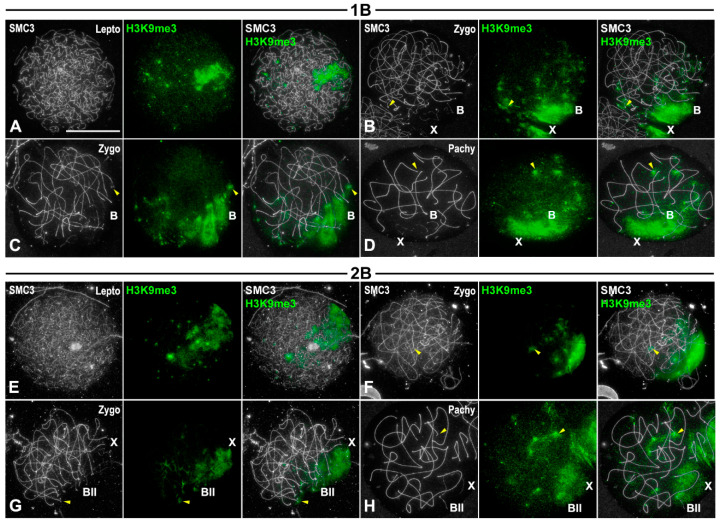
H3K9me3 labeling during prophase I stages. Double immunolabelings of SMC3 (white) and H3K9me3 (green) in prophase I spread spermatocytes from individuals with one (**A**–**D**) and two B1 chromosomes (**E**–**H**). The positions of the sex chromosome (X), the B1 univalent (B) and the B1 bivalent (B_II_) are indicated. (**A**) At leptotene, an intense H3K9me3 signal was visible in a particular nuclear region and discrete accumulations appeared in the rest of the nucleus. (**B**–**D**) From zygotene (**B**,**C**) and up to pachytene (**D**), the large H3K9me3 labeling was maintained and was located in the regions occupied by the X and B1 chromosomes. Additional H3K9me3 signals were positioned at some ends of the SMC3 axes of autosomal bivalents (yellow arrowheads). (**E**–**H**) In individuals carrying two B1 chromosomes, the H3K9me3 labeling was identical to that described above. The large signal corresponded to the positions of the X chromosome and the B1 bivalent. Moreover, some ends of autosome SMC3 axes presented bright foci (yellow arrowheads). Bar in (**A**), 10 µm.

**Figure 5:**
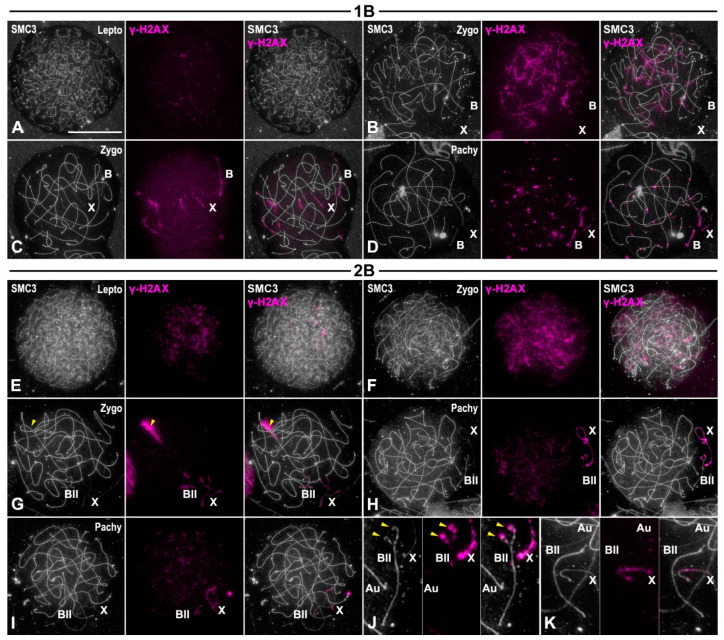
γ-H2AX labeling during prophase I stages. Double immunolabelings of SMC3 (white) and γ-H2AX (purple) in prophase I spread spermatocytes from individuals with one B1 (**A**–**D**) and two B1 chromosomes (**E**–**K**). The positions of the sex chromosome (X), the B1 univalent (B), the B1 bivalent (B_II_) and autosomes (Au) are indicated. (**A**) γ-H2AX labeling became visible at leptotene as discrete flares over the unpaired SMC3 axes. (**B**) At early zygotene, γ-H2AX labeling was only observable over the yet unpaired SMC3 axes of the autosomal bivalents. Both the X and B1 chromosomes did not exhibit γ-H2AX signal. (**C**) By late zygotene, as DSBs repair proceeded, γ-H2AX was restricted to the last unpaired regions of the SMC3 autosomal axes and covered the unpaired SMC3 axes of the X and B1. (**D**) In early/mid pachytene, discrete γ-H2AX foci were positioned over the trajectories of the fully paired autosomal SMC3 axes. In contrast, the X and B1 univalents presented γ-H2AX ribbons covering their single SMC3 axes. (**E**) In individuals with two B1 chromosomes, γ-H2AX labeling become visible at leptotene. (**F**) By early zygotene, γ-H2AX labeling spread over the nucleus, covering unpaired autosomal SMC3 axes. (**G**) At late zygotene, γ-H2AX distribution was limited to the last unpaired regions of the SMC3 axes of autosomes and the B1 bivalent and over the single unpaired axis of the X chromosome. (**H**,**I**) Early and mid-pachytene spermatocytes presented γ-H2AX labeling as foci over paired SMC3 axes at autosomal bivalents and as a more continuous ribbon over the unpaired SMC3 axis of the X chromosome. (**J**,**K**) 300% magnifications of the nuclear region occupied by the X and B1 bivalent during early (**J**) and mid-pachytene (**K**). The B1 bivalent appeared labeled by γ-H2AX over the unpaired ends of the SMC3 axes (yellow arrowheads in (**J**)). By late pachytene (**K**), the B1 bivalent was fully paired and there was no γ-H2AX labeling. Bar in (**A**), 10 µm.

We analyzed the transcriptional activity, in both squashed and spread spermatocytes, of individuals bearing either one or two B1 chromosomes localizing RNA polymerase II phosphorylated at serine 2 (pRNApol II), as a cytological indicator of transcription (Figure 3), K9H3me3 as a marker of heterochromatin (Figure 4) and γ-H2AX (Figure 5) as a maker of DNA damage. The latter two assays showed the location of histone epigenetic modifications associated with chromosome inactivation.

pRNApol II labeling increased from leptotene up to pachytene and covered most of the chromatin (Figure 3). Labeling in the autosomes was extremely faint at the ends of the SMC3 axes, likely representing telomeric and/or pericentromeric heterochromatin (Figure 3B–H) [28]. On the contrary, there was an extreme reduction in pRNApol II labeling over the X, B1 univalents and the B1 bivalents (Figure 3B–H). To compare the intensity of pRNApol II labeling among X, B1 chromosomes and autosomes, we calculated the intensity ratio between pRNApol II/DAPI labeling found over X/B1 chromosomes and autosomes (Supplementary Graph S1B). In zygotene spermatocytes from 0, 1 or 2 B1 individuals, low pRNApol II labeling was found over the X and B1 chromosomes. Interestingly, no statistical differences were found for the pRNApol II/DAPI ratio regardless of the number of B1 chromosomes (Tukey’s multiple comparisons test, *p* > 0.998 in every case). Independently of the chromosome constitution of individuals, autosomes in zygotene nuclei presented a higher pRNApol II labeling intensity than that of X and B1 chromosomes (Tukey’s multiple comparisons test, *p* < 0.0075). No statistical differences were found for the fluorescence intensity associated with autosomes in 0, 1 or 2 B1 individuals (Tukey’s multiple comparisons test, *p* > 0.714 in every case). The low intensity of pRNApol II labeling was maintained over the X and B1 chromosomes in pachytene spermatocytes, with no statistical differences among pachytene nuclei with different numbers of B1 chromosomes (Tukey’s multiple comparisons test, *p* > 0.9999 in every case), nor between zygotene and pachytene spermatocytes (Tukey’s multiple comparisons test, *p* > 0.9999 in every case). In contrast, autosomes in pachytene presented a higher rate of pRNApol II labeling than that scored in zygotene (Tukey’s multiple comparisons test, *p* < 0.0327 in every case), as previously reported [28], and no statistical differences were found among individuals based on the number of B1 chromosomes (Tukey’s multiple comparisons test, *p* > 0.7193 in every case). These results suggest that B1 chromosomes maintain a transcriptionally inactive state during the meiotic phases analyzed regardless of their univalent or bivalent condition (Supplementary Graph S1B).

During prophase I, the histone variant H3K9me3, an epigenetic mark associated with heterochromatin [43], marked some autosomal ends and both the X and the B1 chromosomes. In the latter case, the whole chromosome appeared labeled in both univalent (Figure 4A–D) and bivalent conditions (Figure 4E–H). At metaphase I, and during the second meiotic division, H3K9me3 labeling persisted mostly associated with the pericentromeric region of autosomes and all along the X and B1 chromosomes (Supplementary Figure S2). These findings concur with previous observations on the chromosomal locations of C-bands at these stages (Supplementary Figure S1) [31]. Therefore, it can be concluded that the heterochromatinized state of B1 chromosomes is maintained throughout both meiotic divisions.

On the other hand, γ-H2AX labeling became detectable over unpaired SMC3 axes of autosomes at leptotene (Figure 5A,E). During zygotene, γ-H2AX persisted over the unsynapsed autosomal regions, and became detectable on the X and B1 chromosomes (Figure 5B,C,F,G). The univalent B1 chromosome presented a continuous γ-H2AX labeling (Figure 5C), similar to the X chromosome, while in the B1 bivalent, γ-H2AX labeling was restricted to the unpaired regions of the SMC3 axes (Figure 5G). In early/mid-pachytene, γ-H2AX was detected as discrete foci over the SMC3 axes of the fully synapsed autosomes, and as a ribbon covering the chromatin of the X chromosome (Figure 5D,H,I). In individuals with a single B1 chromosome, γ-H2AX formed a ribbon over the B1 chromosome, as found in the X chromosome (Figure 5D). Contrarily, in the B1 bivalent, γ-H2AX gradually disappeared from the B1 chromosomes, as they achieved synapsis, as occurred in autosomes (Figure 5H–K).

To corroborate these results, we also carried out simultaneous immunolocalization of pRNApol II and H3K9me3 (Supplementary Figure S3), and γ-H2AX and H3K9me3 (Supplementary Figure S4). As expected, we found that H3K9me3 and pRNApol II presented a reverse pattern of distribution within a nucleus, from leptotene to pachytene (Supplementary Figure S3A–F). H3K9me3 and γ-H2AX also presented differences regarding their appearance timing over the X and B1 chromosome(s). While H3K9me3 was observable as early as leptotene (Supplementary Figure S4A,D), γ-H2AX was first detected by zygotene (Supplementary Figure S4B,E). Indeed, whereas H3K9me3 labeling was present throughout prophase I, γ-H2AX labeling disappeared from the B1 bivalent as it achieved synapsis. Altogether, these results allow us to conclude that Bs, in either univalent or bivalent condition, remained transcriptionally silenced during male prophase I and that this inactivation could be related to the accumulation of H3K9me3 and γ-H2AX. However, this inactivation does not preclude the full synapsis achievement of the two B1 chromosomes.

### 3.5. Transcriptional Inactivation of B1 Chromosome Initiates in Spermatogonia

**Figure 6:**
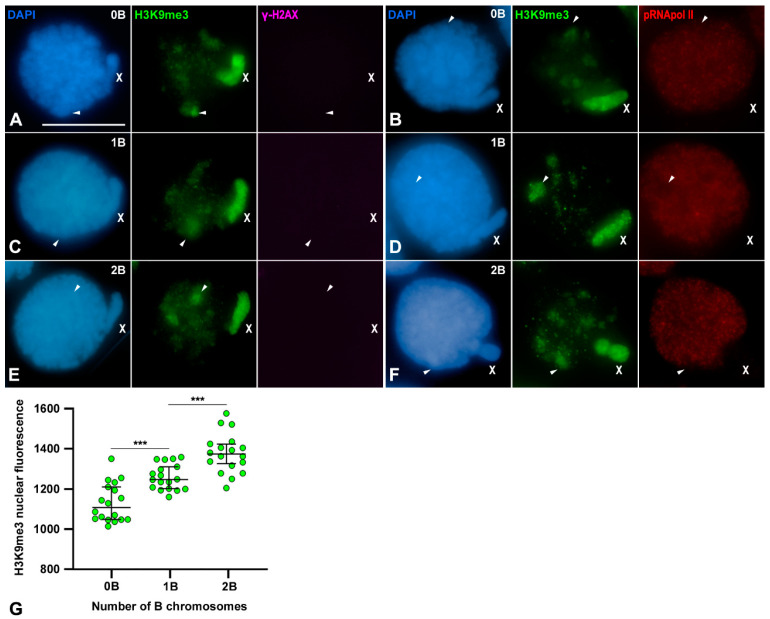
Transcriptional activity in *E. plorans* spermatogonial cells**.** Double immunolabelings of H3K9me3 (green) with either γ-H2AX (purple) or pRNApol II (red) in squashed spermatogonia counterstained with DAPI (blue) from males without (0B), with one (1B) or two (2B) B1 chromosomes**.** The position of the sex chromosome (X) is indicated. (**A**,**B**) Spermatogonia from 0B individuals presented H3K9me3 accumulated at the X chromosome and at certain nuclear regions (white arrowheads). γ-H2AX was not detected and pRNApol II rendered faint labeling over the nucleus, except at those regions labeled with H3K9me3 (white arrowheads). (**C**–**F**) In nuclei of 1B and 2B individuals, H3K9me3 labeled several autosomal regions (white arrowheads), and the X chromosome, but γ-H2AX was not detected. Moreover, H3K9me3 labeling corresponded with regions of reduced pRNApol II labeling. (**G**) Scattered dot plot of the corrected total nuclear fluorescence of H3K9me3 immunolabeling in spermatogonia from individuals presenting 0, 1 or 2 B1 chromosomes. For each class, the individual data (n = 18) and median with 95% CI are depicted. Statistical significance was assessed using an ANOVA test (*p* < 0.0001; ***) and Tukey’s multiple comparisons test. Bar in (**A**), 10 µm.

It has been described that in *E. plorans* males, the silencing of the X chromosome initiates in secondary spermatogonia and is maintained during spermatogenesis [28]. The results presented above suggested that B1 chromosomes were also silenced at the beginning of prophase I (Figure 3 and Figure 4). Therefore, we wondered whether such inactivation might also be generated in secondary spermatogonia. These cells were identified by presenting a rounded nucleus displaying a prominent chromatin protuberance that corresponds to the X chromosome [28,44]. In individuals without B1 chromosomes, the X chromosome chromatin and some autosomal regions, mostly corresponding to pericentromeric and/or telomeric heterochromatin [28], were intensively labeled by H3K9me3 (Figure 6A,B). In these cells, γ-H2AX was not detectable (Figure 6A). Interestingly, the intense H3K9me3 accumulations coincided with nuclear regions presenting either reduced or a complete absence of pRNApol II signals (Figure 6B) [28]. In individuals carrying B1 chromosomes, γ-H2AX foci were not detected (Figure 6C,E) but H3K9me3 accumulations were observed over the X chromosome and at certain nuclear regions with reduced pRNApol II labeling (Figure 6D,F). Although B1 chromosomes were visualized as heteropycnotic bodies after DAPI staining, we could not accurately determine their nuclear position because they did not present a preferential location within the nucleus. Moreover, their location could be confused with that of the heterochromatic pericentromeric regions of the chromosome set. Consequently, we were unable to discern whether the H3K9me3 accumulations undoubtedly corresponded to the B1 chromosomes. To bypass this impediment, we further performed a fluorescence intensity quantification of the nuclear H3K9me3 labeling in spermatogonia with and without B1 chromosomes. We found a positive correlation between the level of H3K9me3 labeling intensity and the number of Bs. Thus, the values obtained increased proportionally with the number of B1 chromosomes of each cell (Figure 6G). Therefore, it could be concluded that B1 chromosomes already presented H3K9me3 labeling in secondary spermatogonia, as it was also observed in the X chromosome and some autosomal regions. Consequently, B1 chromosome inactivation, cytologically highlighted by H3K9me3 accumulation, was already occurring in secondary spermatogonia and would be constitutively maintained throughout meiosis.

## 4. Discussion

The processes of DNA repair, via meiotic recombination, and the fulfillment of synapsis of homologous chromosomes during prophase I are closely related to transcription reactivation in eutherians [8,9,10,12,13]. Thus, unsynapsed chromosomal regions are inactivated by the MSUC mechanism [15,16]. Similarly, sex chromosomes in males with extensive unsynapsed regions undergo a particular form of MSUC termed MSCI [17,18,19]. Although MSCI has been considered one of the critical processes for the success of meiosis in mammalian males [45,46], its evolutionary preservation is far from clear. For instance, in birds and butterflies, where the sex chromosome system is ZW for females and ZZ for males, there is no evidence for MSCI in either synapsed or unsynapsed ZW chromosomes of chicken [47]. Indeed, the ZW bivalent of birds is fully synapsed at pachytene [48,49], and in the lampbrush configuration, the loops were covered with ribonucleoproteins, indicating transcriptional activity [50]. On the other hand, in the flour moth *E. kuehniella* and the silkworm *B. mori*, the Z and W chromosomes are completely non-homologously synapsed at pachytene [37,51,52]. In females of these species, Z and W chromosomes display transcriptional activity at post-pachytene stages [27]. Grasshopper species, with sex chromosome system of the type XX for females and XO for males, contributed to the enlargement of this landscape. Thus, the X chromosome in spermatocytes of the grasshopper *E. plorans* was devoid of the transcriptional marker *p* pRNApol II. The absence of transcription is accompanied by the accumulation of two repressive epigenetic modifications, H3K9me3 and γ-H2AX [28], and the absence of H3K9ac, which is a marker of inactivated chromosome regions [53]. However, the inactive status and the facultative heterochromatinization of the X chromosome likely started in the secondary spermatogonia, well before the beginning of meiosis [28]. This suggests that some of the inactivation features displayed by the X chromosome are not exclusively dependent on its behavior during meiosis, and that perhaps other pre-meiotic factors can be also important for this inactivation process. Here, we provide new evidence on this topic through the study of the behavior of supernumerary B1 chromosomes that presumably derived from the X chromosome [36,54,55,56]. Our results partially challenge some of the assumptions of the MSCU model and reinforce the idea that inactivation of some specific chromosomes might be dependent, at least in part, on events occurring in pre-meiotic cells.

### 4.1. B1 Chromosomes Remain Inactive During Prophase I Regardless of Whether They Complete Synapsis or Not

One of the most remarkable findings of this study is the fact that not only do the X or B1 univalent appear to be subjected to MSUC, but the B1 bivalent does as well. This clearly indicates that in this case transcription and synapsis are not correlated. In *E. plorans* individuals presenting two B1 chromosomes, these chromosomes commonly formed a bivalent with complete synapsis (Figure 1H–J). Indeed, these bivalents showed correct meiotic recombination progression, including the formation of a single chiasma (Figure 2D–I), and accurate chromosomal segregation at anaphase I and anaphase II (Supplementary Figure S1I–P). On these grounds, it would be expected to find transcriptional activity of B1 bivalent throughout prophase I, similar to autosomal ones. Moreover, pRNApol II labeling was detected along autosomes when they still presented extensive unsynapsed regions and γ-H2AX at their chromatin. On the contrary, the B1 bivalent remained inactive even at late pachytene spermatocytes without detectable γ-H2AX labeling associated. These results indicate that transcriptional activity in *E. plorans* spermatocytes might not be tightly linked to synapsis and DNA repair progression. Two sets of our results point in that direction. First, transcription can resume before synapsis and DNA repair have been completed (Figure 3 and Figure S3). The coexistence of transcription and γ-H2AX labeling is especially interesting since the latter is considered the hallmark of MSUC. Second, completing synapsis and DNA repair does not invariably lead to transcription activation (Figure 3 and Figure S3). Thus, other uncharacterized factors may contribute to the B1 transcriptional inactivation. H3K9me3 is an epigenetic mark that has repeatedly been associated with heterochromatin status and meiotic transcription inactivation [6,43]. Of note, we found H3K9me3 to be present over chromosomal regions with reduced amount, or absence, of pRNApol II labeling: the pericentromeric regions of autosomes and whole X and B1 univalents or bivalents (Figure 3, Figure 4 and Figure S3).

Previous studies in mammals have indicated that spermatocytes enter meiosis presenting a distinctive set of epigenetic marks, including H3K9me3 [10]. The broad presence of H3K9me3 over the chromatin of leptotene and zygotene spermatocytes was accompanied by undetectable pRNApol II labeling [6,10]. Moreover, the inability to properly produce and/or process DSBs or to complete synapsis in mouse defective models is accompanied by the persistence of H3K9me3 and the aberrant regulation of transcription in spermatocytes [6]. In the case of *E. plorans* prophase I spermatocytes, the H3K9me3 epigenetic mark might also promote transcriptional inactivation but its more discrete and specific location, at some chromosomal ends of autosomes and over the X and B1 chromosomes, could permit the overall active state of the nucleus. The existence of ten-coding genes in the B24 chromosome, another B variant present in *E. plorans*, which were transcriptionally active in somatic cells, has been reported [57]. However, former experiments using tritiated uridine in grasshoppers and rodents already suggested that the B and X chromosomes might be silenced in meiosis [58,59]. In this context, it is important to consider that the present study has been carried out at the cytological level. Thus, although the chromatin of X and B1 chromosomes mainly remained silenced in *E. plorans* spermatocytes, the transcription of some DNA sequences from the B1 cannot be completely ruled out. Likewise, although the inactivation of the X chromosome in female somatic cells of mammalian species is cytologically associated with heterochromatin marks and reduced presence of RNA polymerase II [60], there are several sequences (i.e., the escapees) known to be transcribed in the inactivated X chromosome [61]. Similarly, several X and Y chromosome-linked genes have been reported to be active in early prophase I stages in mammalian males [11].

### 4.2. X and B1 Chromosomes Enter Meiosis in a Pre-Inactivated State

Another relevant finding of our study is that X and B1 chromosomes, contrarily to autosomes, seem to be already transcriptionally silent in spermatogonia prior to the beginning of meiosis (Figure 6) [28]. The specific pre-meiotic inactivation of these chromosomes in *E. plorans* might denote the existence of certain, yet undiscovered, mechanisms directing their silencing in these cells. Accordingly, at the onset of meiosis X and B1 chromosomes appeared as heteropycnotic positive chromatin bodies and their heterochromatinization is maintained during prophase I. Our data indicate that the accumulation of the repressive epigenetic histone modification H3K9me3 over both chromosomes in spermatogonia, which is maintained during prophase I, might direct their inactivation program in spermatocytes. As previously mentioned, in male mice, the silencing of sex chromosomes during prophase I might reflect their non-reactivation, from a previous repressed state in early meiosis, rather than their specific inactivation during early pachytene. Interestingly, H3K9me3 was postulated to define the initial steps of sex chromosomes’ inactivation at the onset of meiosis [10]. However, in *E. plorans,* the specific inactivation of X and B1 chromosomes is already stablished in spermatogonia. It must be noted that in *E. plorans* populations, it has been postulated that B chromosomes originated from the X chromosome [54,55,56]. The main DNA components of B chromosomes are a 180 bp tandem repeat and 45S rDNA, which can also be found in autosomes and the X chromosome. However, the disposition and order of these DNA sequences in the B1 chromosome are coincident with those specifically found at the X chromosome [54]. Consequently, the similar DNA content and organization of X and B1 chromosomes might prompt a specific chromatin composition, regulated by the presence of specific epigenetic histone modifications, directing their behavior during meiosis. Likewise, previous reports indicated that X and B1 condensed meiotic chromosomes also shared different characteristics of chromatin organization that deviated from the pattern shown by autosomes. In metaphase I, X and B1 univalents are the only chromosomes of the complement of this species showing separated silver-stained chromatid axes, except at centromere and telomere regions [36]. Moreover, these chromosomes presented a zigzagged distribution of the cohesin subunit SMC3 in metaphase I at their interchromatid domain, a pattern distinctively different from that shown by autosomal bivalents [41]. Additionally, H3K9me3 was only associated with the pericentromeric chromatin of autosomal bivalents but covered the chromatin of both X and B1 chromosomes [41]. Overall, the data indicate that the similarities between X and B1 chromosomes are not restricted to their DNA content but also include their chromatin composition and arrangement, their chromosome structure and dynamics, and their behavior during meiosis.

In summary, the bulk of our data contribute to the growing evidence that indicates that, in different species, there are diverse relationships among the specific transcriptional program of prophase I spermatocytes, the presence of meiotic or pre-meiotic chromatin epigenetic modifications, recombination progression and synapsis achievement. Expanding these studies to a variety of wild species bearing different sex chromosomal systems, particular synaptic patterns, or presenting supernumerary chromosomes could contribute to strengthen our knowledge on the topic and its evolutionary history.

## Data Availability

Data are contained within the article and supplementary materials.

## Supplementary Materials

The following supporting information can be downloaded at: https://www.mdpi.com/article/10.3390/genes15121512/s1, Figure S1: Meiotic behavior of B1 chromosomes in *E. plorans* spermatocytes revealed by C-banding. Squashed spermatocytes with one (A–H) or two (I–P) B1 chromosomes after C-banding and DAPI counterstaining. The positions of the sex chromosome (X), a B1 chromosome (B) and a B1 bivalent (BII) are indicated. (A–C) In leptotene (A), zygotene (B) and pachytene (C) spermatocytes discrete DAPI-positive chromosome regions corresponding to autosomal pericentromeric heterochromatin were observed (yellow arrowheads). Additionally, the X and B1 chromosomes showed bright staining, and were closely associated within the nuclear periphery. (D) Diplotene/diakinesis nucleus containing 11 chiasmatic bivalents and the X and B1 chromosomes as univalents. (E) Metaphase I in which B1 appears unaligned at the metaphase plate (top). Bottom: 300% magnification of an autosomal bivalent with a distal chiasma (left) and the X (middle) and B1 (right) chromosomes. (F) During anaphase I, complete chromosomes, including X and B1, segregate to cell poles. (G) Metaphase II containing 13 chromosomes: 11 autosomes plus the X and the B1. (H) During anaphase II, sister chromatids segregate to opposite poles. (I–K) Nuclei of a male carrying two B1 chromosomes in leptotene (I), zygotene (J) and pachytene (K) presenting discrete DAPI-positive regions at autosomal pericentromeric heterochromatin (yellow arrowheads). In leptotene the two B1 could be identified as two large heteropycnotic masses close to the X chromosome, whereas during zygotene and pachytene they became arranged into a bivalent (BII), that was recognizable as a single huge heteropycnotic body (J,K). (L) In diplotene/diakinesis the B1 bivalent (BII) still appeared heteropycnotic. (M) Metaphase I with the B1 bivalent (BII) aligned at the metaphase plate (top). B1 bivalent invariably presented a single chiasma (bottom) that could present a proximal (left), interstitial (middle) or distal location (right). (N) B1 chromosomes segregate to opposite cell poles, as autosomes, during anaphase I. (O) Metaphase II displaying 11 autosomes, the X and one B1 chromosome. (P) During anaphase II, sister chromatids migrate to opposite poles. Bar in (A), 10 µm. Figure S2. H3K9me3 distribution in condensed meiotic chromosomes. Immunolabeling of H3K9me3 (green) in squashed spermatocytes counterstained with DAPI (blue) from *E. plorans* individuals with one B1 (A–D) or two B1 chromosomes (E–J). The positions of the sex chromosome (X), the B1 chromosome (B) and the B1 monochiasmate bivalent (BII) are indicated. (A–D) Metaphase I showing a single B1 chromosome and 300% magnifications of an autosomal bivalent (B), the X (C) and the B1 (D) chromosomes. H3K9me3 labeled the pericentromeric region of autosomes (A,B). The entire chromatin of the X chromosome was covered by H3K9me3 (C). The B1 chromosome also presented H3K9me3 at its entire length including the heterochromatic region close to the centromere (D). (E–H) Metaphase I with a monochiasmatic B1 bivalent (BII) and 300% magnifications of different B1 bivalents. The H3K9me3 centromeric band found in the B1 chromosomes demonstrated that the single chiasma could occur in proximal (F), distal (G) and interstitial regions (H). (I) Metaphase II and (J) anaphase II displayed H3K9me3 labeling at the pericentromeric region of autosomes and over the whole chromatids of the X and B1 chromosomes. Bar in (A), 5 µm. Figure S3. pRNApol II and H3K9me3 distribution during prophase I stages. Double immunolabeling of pRNApol II (red) and H3K9me3 (green) in squashed spermatocytes counterstained with DAPI (blue) from E. plorans individuals with one B1 (A–C) or two B1 chromosomes (D–F). The positions of the sex chromosome (X), the B1 chromosome (B) and the B1 bivalent (BII) are indicated. Note that the X and B1 are heteropycnotic positive chromatin bodies after DAPI staining in prophase I. (A–C) Leptotene (A), zygotene (B) and pachytene (C) spermatocytes with a single B1 chromosome. The intensity of pRNApol II labeling increased as prophase I progressed, but it did not cover those nuclear regions labeled by H3K9me3. The regions with reduced pRNApol II and enriched H3K9me3 labelings mainly corresponded to the chromatin of the X, the B1 chromosomes and to some autosomal heterochromatic regions (yellow arrowheads). (D–F) Leptotene (D), zygotene (E) and pachytene (F) spermatocytes with two B1 chromosomes. pRNApol II and H3K9me3 labeling was identical to that described above. Note that the positions of the regions and chromosomes without pRNApol II labeling and those showing bright H3K9me3 signals coincide. Bar in A, 10 µm. Figure S4. H3K9me3 and γ-H2AX distribution during prophase I. Double immunolabeling of H3K9me3 (green) and γ-H2AX (purple) in squashed spermatocytes counterstained with DAPI (blue) from *E. plorans* individuals with one B1 (A–C) or two B1 chromosomes (D–F). The positions of the sex chromosome (X), the B1 chromosome (B) and the B1 bivalent (BII) are indicated. Note that the X and B1 are heteropycnotic positive chromatin bodies after DAPI staining in prophase I. (A) At leptotene, H3K9me3 labeling marked the X and B1 chromosomes and some autosomal heterochromatic regions (yellow arrowheads). γ-H2AX appeared scattered through the nucleus. (B) During zygotene the H3K9me3 labeling pattern was identical to that described above, whereas γ-H2AX was found in autosomes, X and B1 chromosomes. (C) At pachytene, the distribution of the H3K9me3 labeling remained invariable, but γ-H2AX was found as discrete foci over autosomes and conforming a ribbon over the chromatin of both the X and B1 chromosomes. (D–F) Leptotene (D), zygotene (E) and pachytene (F) spermatocytes with two B1 chromosomes. H3K9me3 labeling is identical to that shown in individuals with one B1 chromone. γ-H2AX labeling was also similar to that exhibited in individuals with one B1 chromosome with a significant difference. B1 chromosomes behave as the autosomes so that they did not show γ-H2AX signals when they became fully synapsed. Bar in A, 10 µm. Supplementary Graph S1. (A) Box whiskers plot analyses of RAD51 foci number per bivalent in E. plorans zygotene and pachytene spermatocytes presenting two B1 chromosomes. The number of RAD51 foci in zygotene and pachytene spermatocytes was initially scored in 10 different cells from individuals presenting two B1 chromosomes (n = 3). No statistical differences were found among individuals in zygotene (ANOVA test, *p* = 0.9916) nor pachytene (ANOVA test, *p* = 0.9983) spermatocytes, and data from different individuals were grouped. The average number of RAD51 foci found in zygotene spermatocytes (blue) were 30.17 and 6.4 for autosomal (Au) and the B1 bivalents, respectively. In pachytene spermatocytes (yellow) the mean number of RAD51 foci were 5.17 and 1.63 at autosomal (Au) and the B1 bivalents, respectively. Statistical differences were found between the number of RAD51 foci located at autosomal and the B1 bivalents in zygotene (unpaired two-tailed *t*-test, *p* < 0.0001; ****), and pachytene (unpaired two-tailed *t*-test, *p* < 0.0001; ****) spermatocytes. For each class, whiskers indicate the minimum to maximum values, first and third quartiles are depicted by the box, and horizontal lines in the boxes indicate the median value. (B) Scattered dot plot of the ratio between pRNApol II and DAPI fluorescence intensity (arbitrary units; A.u.). The intensity of DAPI and pRNApol II staining was scored in zygotene (n = 5) and pachytene (n = 5) nuclei from spermatocytes of individuals presenting 0 (blue dots), 1 (orange dots) and 2 B1 chromosomes (purple dots). We empirically estimated the nuclear region occupied by the X chromosome in 0B1 individuals (X), the X and B1 chromosomes in 1B1 individuals (X+1B) or by the X and two B1 chromosomes in 2B1 individuals (X+2B) in a given nucleus. DAPI and pRNApol II staining intensity were measured and the same selection was placed over the nuclear region occupied by autosomes (Au) to repeat measurements in the same nucleus. After obtaining the corrected total fluorescence for each region of interest, the ratio between pRNApol II and DAPI was calculated. To facilitate the visualization of data, no statistical comparisons are shown in the graphic. See text for further details.

## References

[1] Patel L., Kang R., Rosenberg S.C., Qiu Y., Raviram R., Chee S., Hu R., Ren B., Cole F., Corbett K.D. (2019). Dynamic reorganization of the genome shapes the recombination landscape in meiotic prophase. Nat. Struct. Mol. Biol..

[2] Vara C., Paytuvi-Gallart A., Cuartero Y., Le Dily F., Garcia F., Salva-Castro J., Gomez H.L., Julia E., Moutinho C., Aiese Cigliano R. (2019). Three-Dimensional Genomic Structure and Cohesin Occupancy Correlate with Transcriptional Activity during Spermatogenesis. Cell Rep..

[3] van der Heijden G.W., Derijck A.A., Posfai E., Giele M., Pelczar P., Ramos L., Wansink D.G., van der Vlag J., Peters A.H., de Boer P. (2007). Chromosome-wide nucleosome replacement and H3.3 incorporation during mammalian meiotic sex chromosome inactivation. Nat. Genet..

[4] Kota S.K., Feil R. (2010). Epigenetic transitions in germ cell development and meiosis. Dev. Cell.

[5] Lam K.-W.G., Brick K., Cheng G., Pratto F., Camerini-Otero R.D. (2019). Cell-type-specific genomics reveals histone modification dynamics in mammalian meiosis. Nat. Commun..

[6] de la Fuente R., Pratto F., Hernández-Hernández A., Manterola M., López-Jiménez P., Gómez R., Viera A., Parra M.T., Kouznetsova A., Camerini-Otero R.D. (2021). Epigenetic Dysregulation of Mammalian Male Meiosis Caused by Interference of Recombination and Synapsis. Cells.

[7] Monesi V. (1965). Synthetic activities during spermatogenesis in the mouse RNA and protein. Exp. Cell Res..

[8] Almstrup K., Nielsen J.E., Hansen M.A., Tanaka M., Skakkebaek N.E., Leffers H. (2004). Analysis of cell-type-specific gene expression during mouse spermatogenesis. Biol. Reprod..

[9] Shima J.E., McLean D.J., McCarrey J.R., Griswold M.D. (2004). The murine testicular transcriptome: Characterizing gene expression in the testis during the progression of spermatogenesis. Biol. Reprod..

[10] Page J., de la Fuente R., Manterola M., Parra M.T., Viera A., Berrios S., Fernandez-Donoso R., Rufas J.S. (2012). Inactivation or non-reactivation: What accounts better for the silence of sex chromosomes during mammalian male meiosis?. Chromosoma.

[11] da Cruz I., Rodríguez-Casuriaga R., Santiñaque F.F., Farías J., Curti G., Capoano C.A., Folle G.A., Benavente R., Sotelo-Silveira J.R., Geisinger A. (2016). Transcriptome analysis of highly purified mouse spermatogenic cell populations: Gene expression signatures switch from meiotic-to postmeiotic-related processes at pachytene stage. BMC Genom..

[12] Schultz N., Hamra F.K., Garbers D.L. (2003). A multitude of genes expressed solely in meiotic or postmeiotic spermatogenic cells offers a myriad of contraceptive targets. Proc. Natl. Acad. Sci. USA.

[13] Pang A.L., Johnson W., Ravindranath N., Dym M., Rennert O.M., Chan W.Y. (2006). Expression profiling of purified male germ cells: Stage-specific expression patterns related to meiosis and postmeiotic development. Physiol. Genom..

[14] Baarends W.M., Wassenaar E., van der Laan R., Hoogerbrugge J., Sleddens-Linkels E., Hoeijmakers J.H., de Boer P., Grootegoed J.A. (2005). Silencing of unpaired chromatin and histone H2A ubiquitination in mammalian meiosis. Mol. Cell. Biol..

[15] Schimenti J. (2005). Synapsis or silence. Nat. Genet..

[16] Turner J.M., Mahadevaiah S.K., Fernandez-Capetillo O., Nussenzweig A., Xu X., Deng C.X., Burgoyne P.S. (2005). Silencing of unsynapsed meiotic chromosomes in the mouse. Nat. Genet..

[17] Mahadevaiah S.K., Turner J.M., Baudat F., Rogakou E.P., de Boer P., Blanco-Rodriguez J., Jasin M., Keeney S., Bonner W.M., Burgoyne P.S. (2001). Recombinational DNA double-strand breaks in mice precede synapsis. Nat. Genet..

[18] Turner J.M., Aprelikova O., Xu X., Wang R., Kim S., Chandramouli G.V., Barrett J.C., Burgoyne P.S., Deng C.X. (2004). BRCA1, histone H2AX phosphorylation, and male meiotic sex chromosome inactivation. Curr. Biol..

[19] Turner J.M. (2007). Meiotic sex chromosome inactivation. Development.

[20] Henderson S.A. (1963). Differential Ribonucleic Acid Synthesis of X and Autosomes During Meiosis. Nature.

[21] Henderson S.A. (1964). RNA synthesis during male meiosis and spermiogenesis. Chromosoma.

[22] Das N.K., Siegel E.P., Alfert M. (1965). Synthetic Activities During Spermatogenesis in the Locust. J. Cell Biol..

[23] Chu D.S., Shakes D.C. (2013). Spermatogenesis. Adv. Exp. Med. Biol..

[24] Hennig W., Weyrich A. (2013). Histone modifications in the male germ line of Drosophila. BMC Dev. Biol..

[25] Palacios-Gimenez O.M., Marti D.A., Cabral-de-Mello D.C. (2015). Neo-sex chromosomes of Ronderosia bergi: Insight into the evolution of sex chromosomes in grasshoppers. Chromosoma.

[26] de Almeida B.R.R., Noronha R.C.R., da Costa M.J.R., Nagamachi C.Y., Pieczarka J.C. (2019). Meiosis in the scorpion Tityus silvestris: New insights into achiasmatic chromosomes. Biol. Open.

[27] Traut W., Schubert V., Dalikova M., Marec F., Sahara K. (2019). Activity and inactivity of moth sex chromosomes in somatic and meiotic cells. Chromosoma.

[28] Viera A., Parra M.T., Arevalo S., Garcia de la Vega C., Santos J.L., Page J. (2021). X Chromosome Inactivation during Grasshopper Spermatogenesis. Genes.

[29] Viera A., Parra M.T., Rufas J.S., Page J. (2017). Transcription reactivation during the first meiotic prophase in bugs is not dependent on synapsis. Chromosoma.

[30] Henriques-Gil N., Santos J.L., Arana P. (1984). Evolution of a complex B-chromosome polymorphism in the grasshopper Eyprepocnemis plorans. Chromosoma.

[31] Henriques-Gil N., Santos J.L., Giraldez R. (1982). B-chromosome polymorphism and interchromosomal chiasma interference in Eyprepocnemis plorans (Acrididae; Orthoptera). Chromosoma.

[32] Camacho J.P.M., Carballo A.R., Cabrero J. (1980). The B-chromosome system of the grasshopper Eyprepocnemis plorans subsp. plorans (Charpentier). Chromosoma.

[33] Camacho J.P., Sharbel T.F., Beukeboom L.W. (2000). B-chromosome evolution. Philos. Trans. R. Soc. London. Ser. B Biol. Sci..

[34] Peters A.H., Plug A.W., van Vugt M.J., de Boer P. (1997). A drying-down technique for the spreading of mammalian meiocytes from the male and female germline. Chromosome Res..

[35] Page J., Suja J.A., Santos J.L., Rufas J.S. (1998). Squash procedure for protein immunolocalization in meiotic cells. Chromosome Res..

[36] Viera A., Calvente A., Page J., Parra M.T., Gomez R., Suja J.A., Rufas J.S., Santos J.L. (2004). X and B chromosomes display similar meiotic characteristics in male grasshoppers. Cytogenet. Genome Res..

[37] Xiang Y., Tsuchiya D., Yu Z., Zhao X., McKinney S., Unruh J., Slaughter B., Lake C.M., Hawley R.S. (2024). Multiple reorganizations of the lateral elements of the synaptonemal complex facilitate homolog segregation in Bombyx mori oocytes. Curr. Biol..

[38] Viera A., Santos J.L., Page J., Parra M.T., Calvente A., Cifuentes M., Gomez R., Lira R., Suja J.A., Rufas J.S. (2004). DNA double-strand breaks, recombination and synapsis: The timing of meiosis differs in grasshoppers and flies. EMBO Rep..

[39] Viera A., Parra M.T., Page J., Santos J.L., Rufas J.S., Suja J.A. (2003). Dynamic relocation of telomere complexes in mouse meiotic chromosomes. Chromosome Res..

[40] Cerro A.L., Santos J.L. (1995). Synapsis in grasshopper bivalents heterozygous for centric shifts. Genome.

[41] Calvente A., Viera A., Parra M.T., de la Fuente R., Suja J.A., Page J., Santos J.L., de la Vega C.G., Barbero J.L., Rufas J.S. (2013). Dynamics of cohesin subunits in grasshopper meiotic divisions. Chromosoma.

[42] Shinohara A., Ogawa H., Ogawa T. (1992). Rad51 protein involved in repair and recombination in *S. cerevisiae* is a RecA-like protein. Cell.

[43] Cowell I.G., Aucott R., Mahadevaiah S.K., Burgoyne P.S., Huskisson N., Bongiorni S., Prantera G., Fanti L., Pimpinelli S., Wu R. (2002). Heterochromatin, HP1 and methylation at lysine 9 of histone H3 in animals. Chromosoma.

[44] Church K. (1979). The grasshopper X chromosome. I. States of condensation and the nuclear envelope at G1, S and G2 of premeiotic interphase and at early meiotic prophase. Chromosoma.

[45] Turner J.M. (2015). Meiotic Silencing in Mammals. Annu. Rev. Genet..

[46] Abe H., Yeh Y.H., Munakata Y., Ishiguro K.I., Andreassen P.R., Namekawa S.H. (2022). Active DNA damage response signaling initiates and maintains meiotic sex chromosome inactivation. Nat. Commun..

[47] Guioli S., Lovell-Badge R., Turner J.M. (2012). Error-prone ZW pairing and no evidence for meiotic sex chromosome inactivation in the chicken germ line. PLoS Genet..

[48] Solari A.J. (1992). Equalization of Z and W axes in chicken and quail oocytes. Cytogenet. Cell Genet..

[49] Pigozzi M.I. (2016). The Chromosomes of Birds during Meiosis. Cytogenet. Genome Res..

[50] Gaginskaya E., Kulikova T., Krasikova A. (2009). Avian lampbrush chromosomes: A powerful tool for exploration of genome expression. Cytogenet. Genome Res..

[51] Weith A., Traut W. (1986). Synaptic adjustment, non-homologous pairing, and non-pairing of homologous segments in sex chromosome mutants of *Ephestia kuehniella* (Insecta, Lepidoptera). Chromosoma.

[52] Marec F., Traut W. (1993). Synaptonemal complexes in female and male meiotic prophase of *Ephestia kuehniella* (Lepidoptera). Heredity.

[53] Cabrero J., Teruel M., Carmona F.D., Jimenez R., Camacho J.P. (2007). Histone H3 lysine 9 acetylation pattern suggests that X and B chromosomes are silenced during entire male meiosis in a grasshopper. Cytogenet. Genome Res..

[54] Lopez-Leon M.D., Neves N., Schwarzacher T., Heslop-Harrison J.S., Hewitt G.M., Camacho J.P. (1994). Possible origin of a B chromosome deduced from its DNA composition using double FISH technique. Chromosome Res..

[55] Cabrero J., Lopez-Leon M.D., Bakkali M., Camacho J.P. (1999). Common origin of B chromosome variants in the grasshopper eyprepocnemis plorans. Heredity.

[56] Cabrero J., Perfectti F., Gomez R., Camacho J.P., Lopez-Leon M.D. (2003). Population variation in the A chromosome distribution of satellite DNA and ribosomal DNA in the grasshopper Eyprepocnemis plorans. Chromosome Res..

[57] Navarro-Dominguez B., Ruiz-Ruano F.J., Cabrero J., Corral J.M., Lopez-Leon M.D., Sharbel T.F., Camacho J.P. (2017). Protein-coding genes in B chromosomes of the grasshopper Eyprepocnemis plorans. Sci. Rep..

[58] Fox D.P., Hewitt G.M., Hall D.J. (1974). DNA replication and RNA transcription of euchromatic and heterochromatic chromosome regions during grasshopper meiosis. Chromosoma.

[59] Ishak B., Jaafar H., Maetz J.L., Rumpler Y. (1991). Absence of transcriptional activity of the B-chromosomes of Apodemus peninsulae during pachytene. Chromosoma.

[60] Collombet S., Rall I., Dugast-Darzacq C., Heckert A., Halavatyi A., Le Saux A., Dailey G., Darzacq X., Heard E. (2023). RNA polymerase II depletion from the inactive X chromosome territory is not mediated by physical compartmentalization. Nat. Struct. Mol. Biol..

[61] Loda A., Collombet S., Heard E. (2022). Gene regulation in time and space during X-chromosome inactivation. Nat. Rev. Mol. Cell Biol..

