# Supplementary figures and images for: B Chromosome Transcriptional Inactivation in the Spermatogenesis of the Grasshopper Eyprepocnemis plorans
Source: Genes (Basel). 2024 Nov 25;15(12):1512. doi: 10.3390/genes15121512 (PMC11675390; doi:10.3390/genes15121512)

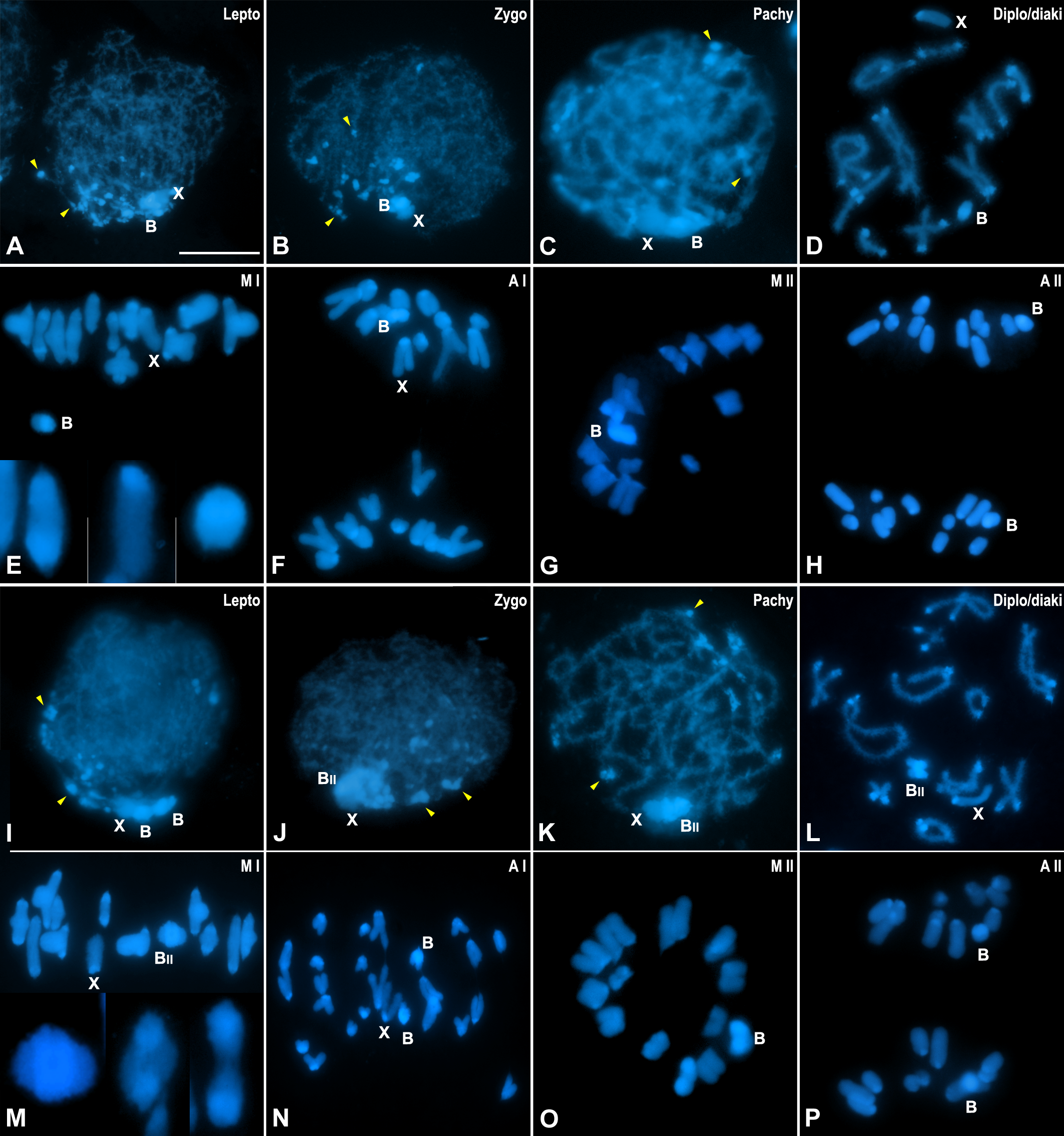

Supplement: Supplementary file 1 [file genes-15-01512-s001.zip › Supplementary figure S1.tif]

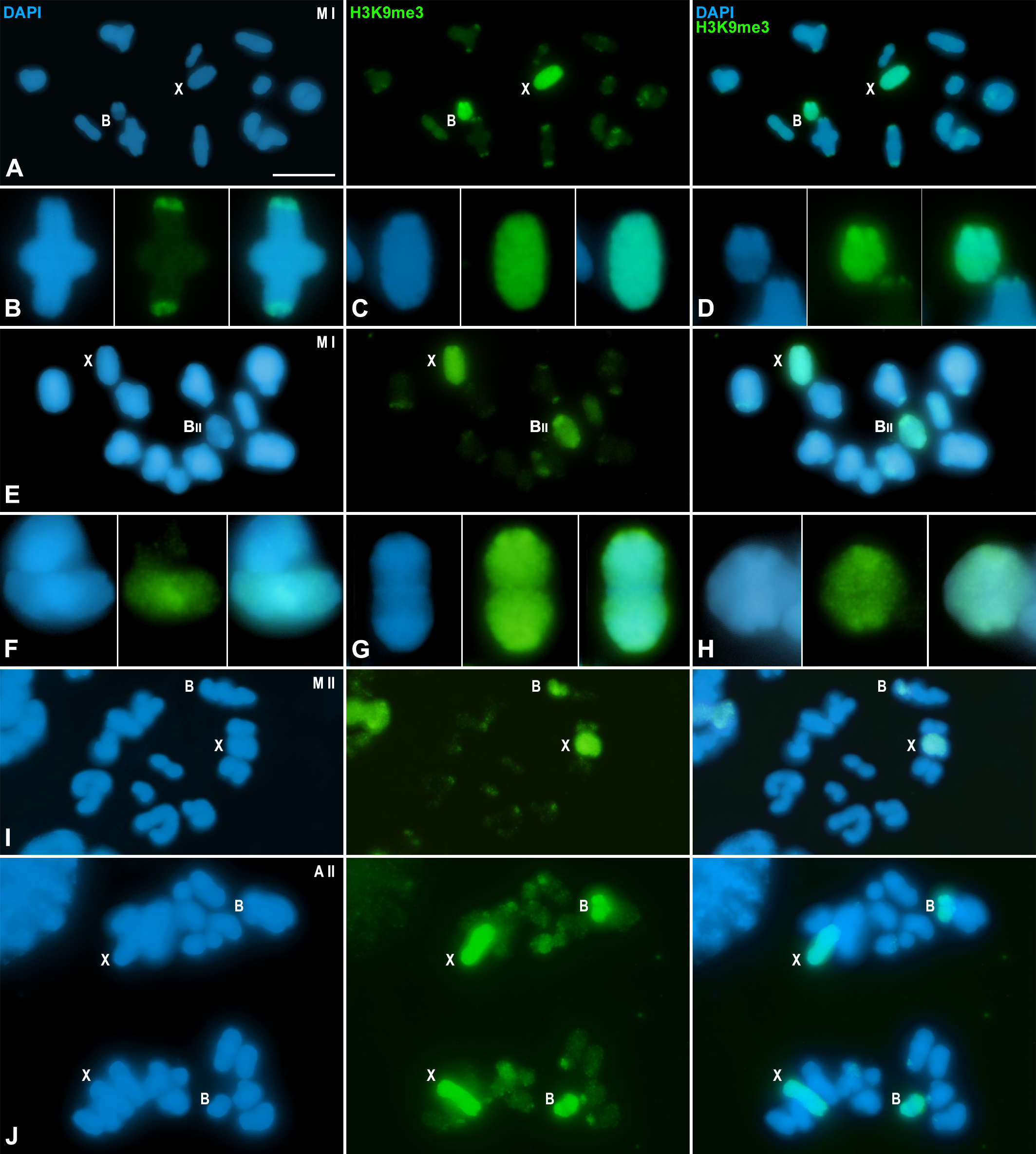

Supplement: Supplementary file 1 [file genes-15-01512-s001.zip › Supplementary figure S2.tif]

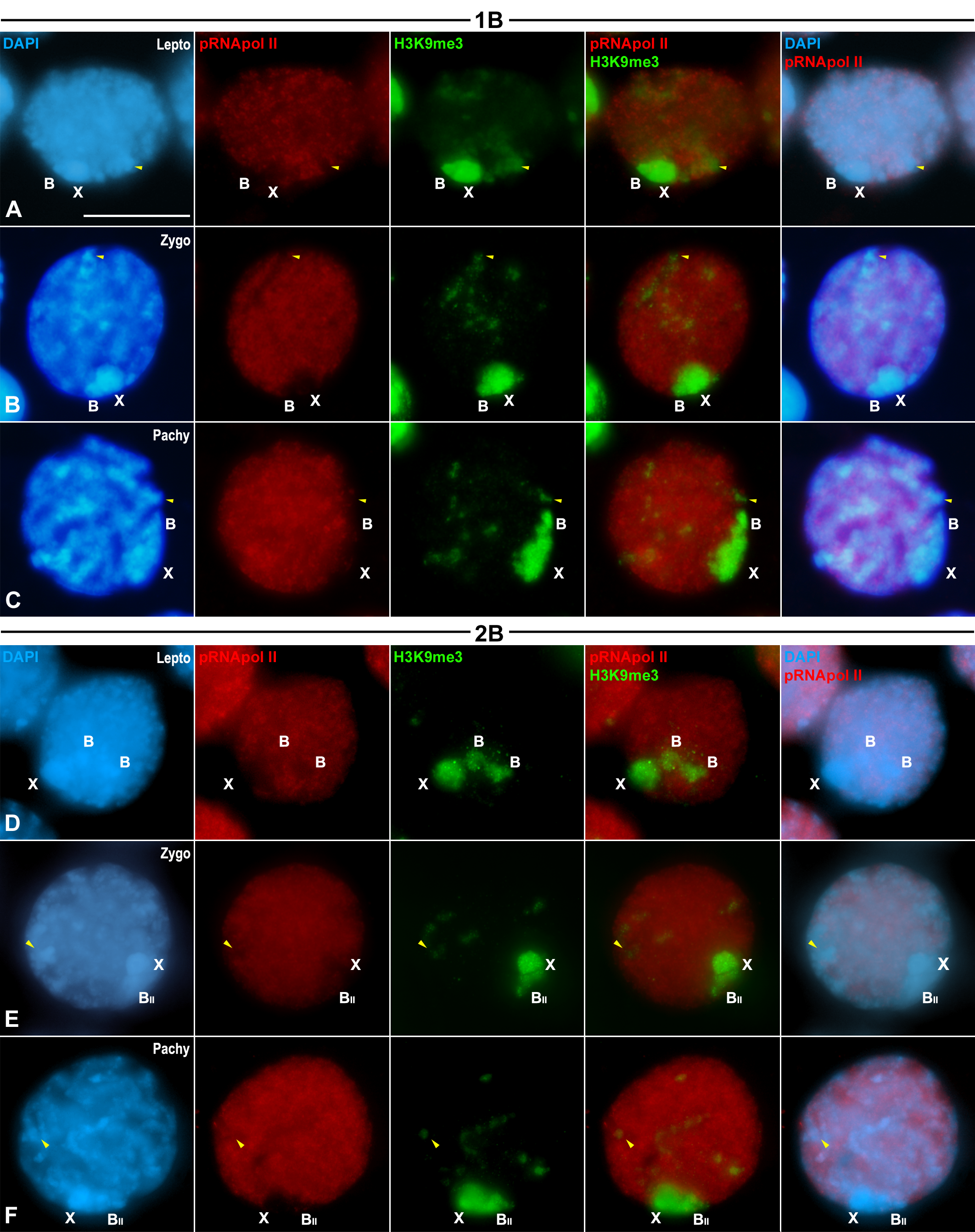

Supplement: Supplementary file 1 [file genes-15-01512-s001.zip › Supplementary figure S3.tif]

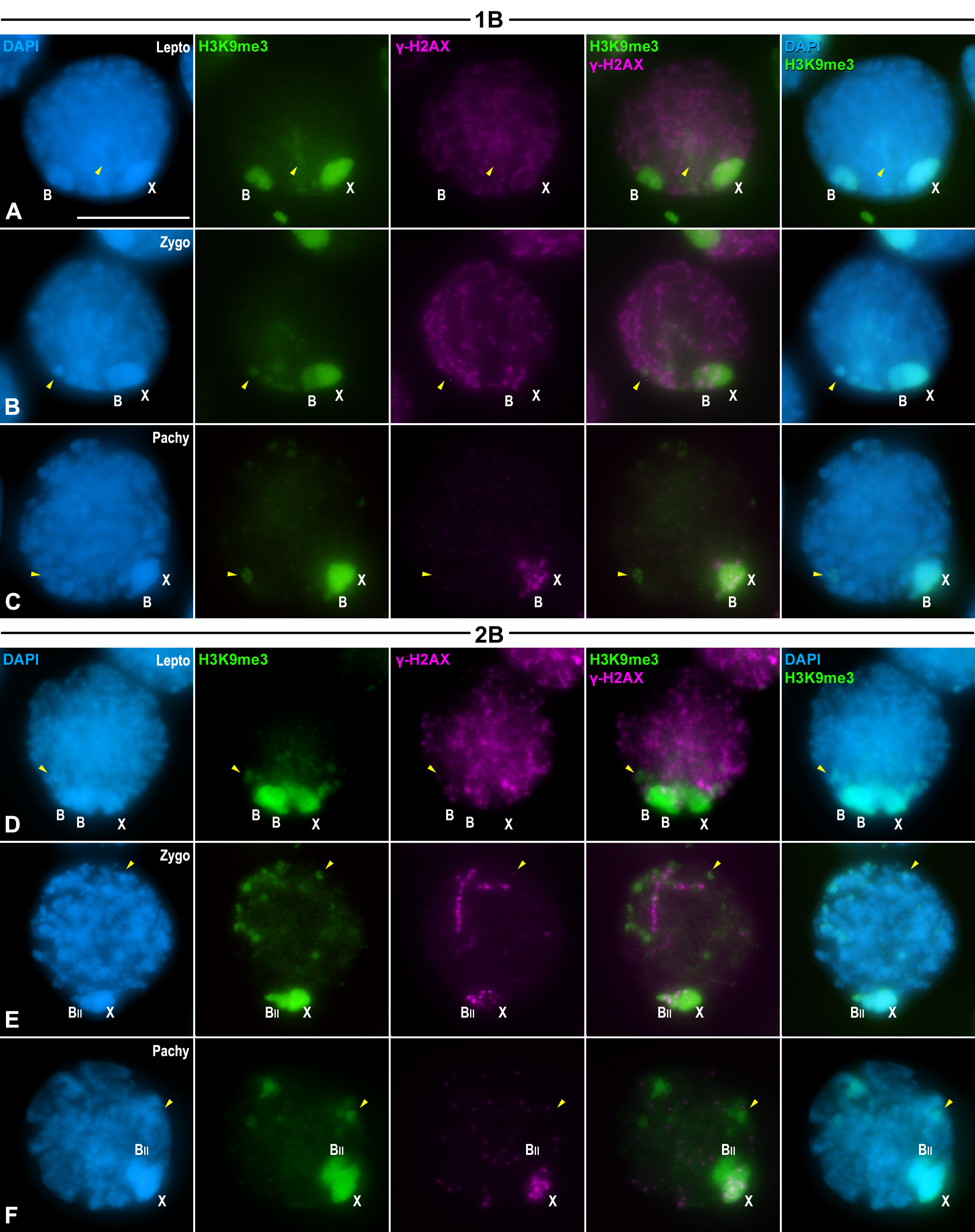

Supplement: Supplementary file 1 [file genes-15-01512-s001.zip › Supplementary figure S4.tif]

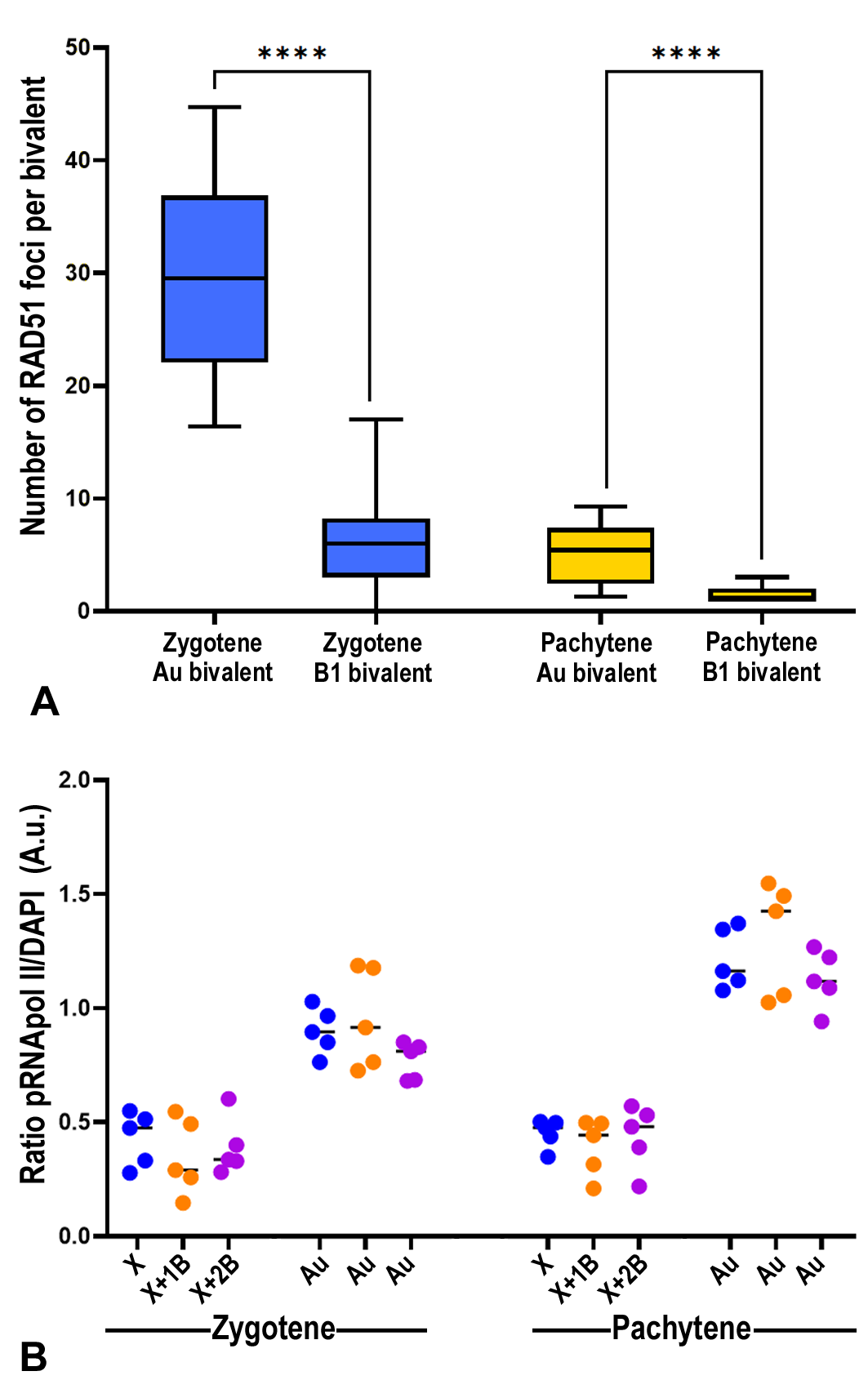

Supplement: Supplementary file 1 [file genes-15-01512-s001.zip › Supplementary graph S1.tif]
